# Eosinophilia and the Elusive Skin Condition, Spongiotic Dermatitis

**DOI:** 10.7759/cureus.91131

**Published:** 2025-08-27

**Authors:** Regina J McPherson, Juan Ramon Santos Rivera, Ilya Fonarov

**Affiliations:** 1 Medicine, Florida International University, Herbert Wertheim College of Medicine, Miami, USA; 2 Internal Medicine, Ponce Health Sciences University, Ponce, PRI; 3 Internal Medicine, Jackson Memorial Hospital, Miami, USA

**Keywords:** cutaneous hypersensitivity, dermatopathology, hyper-eosinophilia, inflammatory dermatoses, spongiotic dermatitis

## Abstract

Chronic eosinophilia with cutaneous involvement presents a diagnostic challenge, as the differential diagnosis is very broad, including eosinophilic dermatoses, hypersensitivity reactions, autoimmune diseases, and infections. Spongiotic dermatitis (SD) is a histopathological pattern commonly observed in inflammatory skin conditions. It is a non-specific finding that requires clinical correlation. This case describes a 68-year-old female with a five-year history of chronic eosinophilia who presented with acute right leg pain, swelling, and a diffuse pruritic rash covering >90% of her body surface area. She reported recurrent flares of erythematous plaques with bilateral eye swelling and had a history of recent international travel. Laboratory testing revealed marked eosinophilia, elevated IgE levels, and increased inflammatory markers, while a punch biopsy demonstrated SD with eosinophilic infiltration. Given the histopathologic findings and systemic involvement, multiple etiologies were considered. This report highlights the diagnostic complexity of SD in the context of chronic eosinophilia, emphasizing the importance of distinguishing between mimicking conditions through a thorough clinical and histopathologic evaluation.

## Introduction

Spongiotic dermatitis (SD) is the most common histopathological reaction pattern observed in inflammatory skin conditions. It is defined by intercellular epidermal edema (spongiosis), which may lead to intraepidermal vesicle formation when severe [1]. This pattern is seen in a broad range of disorders and is considered histologically non-specific, often requiring correlation with clinical features for an accurate diagnosis [2]. It typically progresses through acute, subacute, and chronic stages. In acute phases, pronounced spongiosis dominates; in later stages, features such as parakeratosis, acanthosis, and hyperkeratosis become more prominent [3].

The clinical presentation of SD varies widely, ranging from erythematous papules and vesicles to lichenified plaques in chronic cases. Common causes include atopic dermatitis, allergic contact dermatitis, nummular dermatitis, dyshidrotic eczema, seborrheic dermatitis, and pityriasis rosea [3,4]. Although atopic dermatitis is the most frequent etiology, histopathological confirmation may be needed when presentation is atypical [5]. Factors contributing to SD include genetic mutations (e.g., filaggrin deficiency), skin barrier disruption, environmental triggers, and immune dysregulation [5,6].

Less common causes of SD include drug reactions, dermatophytosis, and systemic conditions such as drug reaction with eosinophilia and systemic symptoms (DRESS), eosinophilic cellulitis (Wells syndrome), and hypereosinophilic syndrome (HES) [7-9]. These entities may present with widespread eruptions, often symmetric, pruritic, and morbilliform, sometimes progressing to vesicles or bullae depending on the severity [8]. Because of overlapping features among spongiotic dermatoses, histopathologic evaluation remains essential for narrowing the differential diagnosis and guiding appropriate management [1,2,4]. Therefore, this report presents a case of SD with eosinophilic infiltration in a patient with chronic eosinophilia without a prior definitive diagnosis.

## Case presentation

A 68-year-old woman with a five-year history of chronic eosinophilia of unknown etiology presented to the ER with throbbing right leg/ankle and thigh pain and swelling that started two days before presentation. She reported that two days ago, she woke up with a fever and chills and that throughout the day, her right leg and ankle started to hurt; she noticed progressive swelling that evolved to include her thigh. The patient denied any trauma. She reported a diffuse, itchy rash that flares intermittently and has been present on and off for about five years. Her flares typically involved bilateral eye redness and swelling. She has been on chronic prednisone for symptomatic treatment, as her work-up had not identified the etiology. The patient had recently travelled internationally to Argentina. It is unknown whether the patient had a previous skin biopsy. She denied any new medications, denied exposure to exotic animals or plants, and denied recent infections or vaccinations. She had joint pains, fatigue, nausea/vomiting, diarrhea, night sweats, unintentional weight loss, difficulty swallowing, or oral lesions. She denied any history of tobacco use, alcohol, or illicit drugs, and any family history of autoimmune diseases.

**Figure 1:**
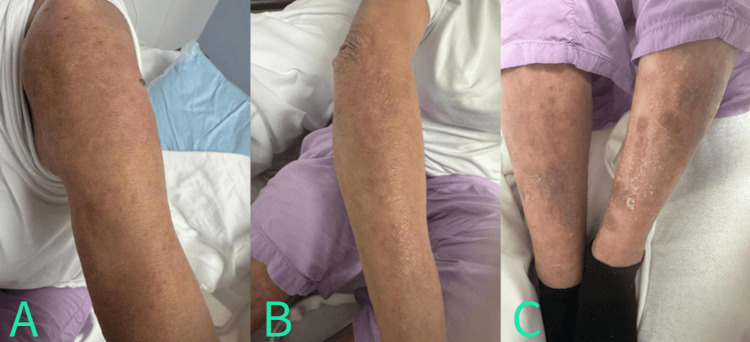
Bilateral hands noted with erythematous flaky rash (A and B). Multiple pink-to-salmon-to-violaceous ill-defined urticarial plaques with hemorrhagic crusting on the bilateral lower extremities (C).

On evaluation, the patient's vital signs were within normal limits. Her physical exam was only impressive for a diffuse rash present on >90% of the total body surface area. Specifically, she had multiple pink-to-salmon-to-violaceous ill-defined urticarial plaques with hemorrhagic crusting on the bilateral lower extremities (Figure 1, panel C). Her bilateral hands were remarkable for an erythematous, flaky rash (Figure 1, panels A and B). Initial laboratory results showed elevated eosinophil absolute counts and percentage, elevated total IgE, and inflammatory markers (Table 1). Other laboratory values were unremarkable, including a negative complete autoimmune panel. A lower extremity Doppler ultrasound was negative for acute deep vein thrombosis (DVT). 

**Table 1:** Relevant laboratory values on admission

Laboratory values	Results	Reference ranges
Blood urea nitrogen	23 mg/dL	7-17 mg/dL
Creatinine	0.90 mg/dL	0.66-1.25 mg/dL
C-reactive protein	4.0 mg/dL	0.0-0.9 mg/dL
Erythrocyte sedimentation rate	67 m/hr	0-30 mm/hr
White blood cell count	10.1 X10(3)/mcL	4.0-10.5 X 10(3)/mcL
Eosinophil (%)	17.5%	0.0-5.0%
Absolute eosinophil	1.77 X 10(3)/mcL	0.10-0.50 X 10(3)/mcL
Total serum immunoglobulin E level	527.81 int_unit/mL	0.00–99.99 int_unit/mL

**Figure 2:**
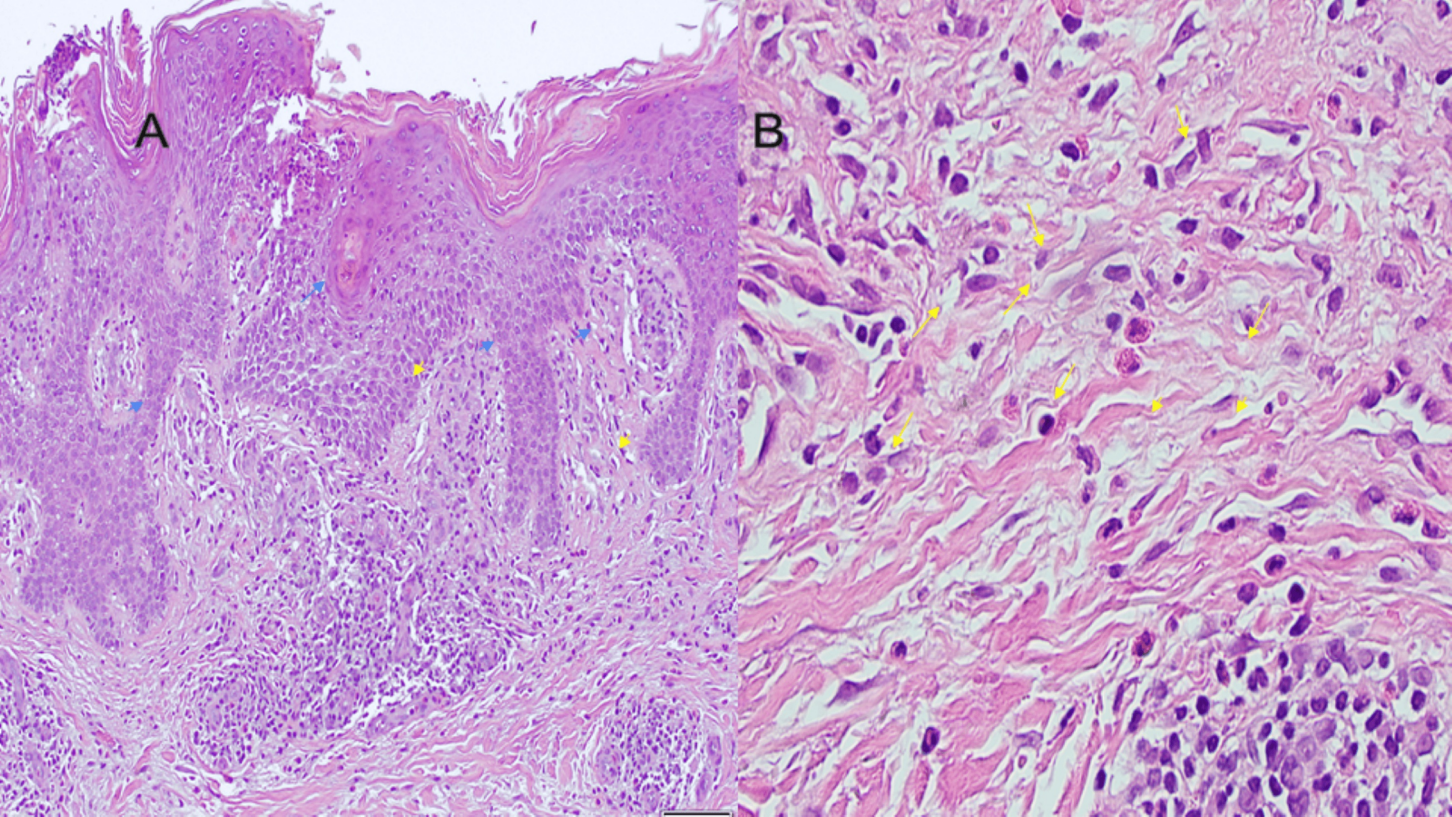
Histopathological image Skin biopsy with foci of spongiosis (blue arrows), irregular acanthosis (blue arrowheads), and overlying parakeratosis and crust. There is a superficial perivascular lymphocytic infiltrate (yellow arrowheads) with abundant eosinophils (yellow arrow). A: Hematoxylin & eosin stain, magnification 10x; B: Hematoxylin & eosin stain, magnification 40x

Initial management included topical clobetasol ointment and oral prednisone for seven days. The differential at the time included eosinophilic cellulitis, urticarial vasculitis, and morbilliform eruption. Dermatology performed a bedside punch biopsy, which showed SD with eosinophils (Figure 2). No evidence of vasculitis was seen. The patient was discharged with a dermatology follow-up in two weeks for further management. At the follow-up, the patient had minimal improvement in her skin lesions and required further treatment with topical agents, and will need long-term follow-up with dermatology. 

## Discussion

This case highlights a presentation of SD with eosinophilic infiltration in a patient with chronic eosinophilia, elevated IgE levels, and extensive cutaneous involvement. While the biopsy confirmed SD, the case underscores the diagnostic challenge of distinguishing SD from other eosinophilic dermatoses, systemic hypersensitivity reactions, autoimmune conditions, and parasitic infections, all of which share overlapping clinical and histopathologic features [1,2,4].

Accurate recognition of SD and its mimics is essential to avoid misdiagnosis and ensure proper treatment. Though SD is typically seen in benign inflammatory dermatoses such as atopic dermatitis, contact dermatitis, or drug-related reactions [1,3], its occurrence in patients with eosinophilia warrants broader consideration of conditions with systemic implications. Atopic dermatitis, for example, is often driven by type 2 inflammation, IgE dysregulation, and barrier defects, factors that may also be seen in DRESS, Wells syndrome, or HES [4,5].

Spongiotic dermatitis is histologically characterized by spongiosis, parakeratosis, and superficial perivascular lymphocytic infiltrates, occasionally with eosinophils [1,4]. However, the clinical appearance of SD is highly variable and may mimic other dermatologic or infectious conditions. This was clearly demonstrated in a recent case by Lewis et al., where a 64-year-old woman with chronic vaginal irritation underwent multiple incorrect treatments before a biopsy confirmed SD. Despite testing negative for multiple infections and having atypical genital findings, the diagnosis remained elusive until histopathology revealed characteristic spongiosis, lymphocytic exocytosis, and superficial inflammation. Following biopsy confirmation, treatment with topical corticosteroids led to symptom resolution [10]. This case emphasizes that SD can present outside typical locations and mimic infectious or autoimmune conditions, reinforcing the critical role of biopsy in diagnosis.

To aid in the differentiation of SD from other eosinophilic skin conditions, a comparative table (Table 2) is provided below. This table outlines key clinical, laboratory, histopathologic, and treatment features of SD, parasitic infections, Wells syndrome, DRESS, and HES. Given the overlapping presentations, this side-by-side comparison may assist clinicians in refining the differential diagnosis, particularly in patients with systemic symptoms or marked eosinophilia.

**Table 2:** Comparison of SD with other eosinophilic dermatoses and mimics, including parasitic infections, Wells syndrome, DRESS, and HES This table summarizes key differences in clinical presentation, eosinophil involvement, systemic features, histopathology, common triggers, and standard treatments to aid in differential diagnosis SD: Spongiotic dermatitis, DRESS: Drug reaction with eosinophilia and systemic symptoms, HES: Hypereosinophilic syndrome

Pathology	Definition/etiology	Eosinophilia	Skin manifestations	Systemic involvement	Histopathology	Common triggers	Treatment	Source
SD	An eczematous reaction pattern characterized by intercellular edema (spongiosis) seen in conditions like allergic contact or atopic dermatitis	Usually minimal; may have mild eosinophils in allergic cases	Pruritic, eczematous lesions that are often vesicular, oozing, or scaling; consistent with an inflammatory reaction	Generally confined to the skin without systemic involvement	Spongiosis with a perivascular lymphocytic infiltrate; occasional eosinophils may be seen	Allergens, irritants, atopic predisposition (e.g., allergic contact dermatitis)	Emollients, trigger avoidance, and topical/systemic corticosteroids. Immunosuppressants in severe cases	[11]
Parasitic infections	Caused by parasites; exposure-related with varied clinical presentations	Often present; the degree may vary depending on the parasite and host response	Varied presentations, e.g., serpiginous (migratory) tracks in cutaneous larva migrans, urticaria, papules, or nodules	Can be limited to the skin (e.g., cutaneous larva migrans) or involve the systemic system	Inflammatory infiltrate rich in eosinophils; granulomatous features can be present in some parasitic infections	Environmental exposure to parasites (e.g., tropical travel, contaminated water/soil)	Antiparasitic agents along with supportive measures	[12]
Wells syndrome	Eosinophilic cellulitis is characterized by recurrent inflammatory, cellulitis-like plaques; often idiopathic or triggered by insect bites or medications	Common in skin lesions (flame figures) and may show peripheral eosinophilia	Edematous, erythematous plaques that can mimic cellulitis; may evolve into vesicles or bullae	Primarily confined to the skin; systemic symptoms are rare	Dermal eosinophilic infiltrate with 'flame figures' (degenerating collagen coated with eosinophilic granule proteins)	Often idiopathic; can be triggered by insect bites, medications, or otherwise unknown	Topical or systemic steroids; in some cases, immunomodulatory therapies	[13,14]
DRESS	Severe drug-induced hypersensitivity reaction with systemic involvement (fever, lymphadenopathy, organ injury)	Typically present as part of the systemic reaction	Morbilliform rash that may progress to exfoliative dermatitis, often with facial edema	Prominent systemic involvement (liver, kidney, hematologic abnormalities, etc.)	Variable; may show interface dermatitis with necrotic keratinocytes and eosinophils	Specific medications such as anticonvulsants, sulfa drugs, allopurinol, etc.	Immediate withdrawal of the offending drug, followed by systemic corticosteroids and supportive care	[15,16]
HES	A disorder defined by persistently high eosinophil counts (>1500 cells/micro liter) leading to end-organ damage; may be idiopathic or secondary to hematologic disorders	Marked, persistent peripheral eosinophilia with tissue infiltration	Variable; may present as urticarial, papular, or nodular rashes; non-specific findings	Frequently involves multiple organs (heart, lungs, gastrointestinal tract, nervous system)	Non-specific tissue eosinophillic infiltrates with evidence of organ damage; not limited to skin	Frequently idiopathic; may be associated with myeloproliferative disorders or other systemic conditions	Systemic corticosteroids are first line; may require cytoreductive therapy or targeted biologics	[17,18]

The key approach in the management of SD is to avoid triggers when identifiable and restore the skin barrier through routine moisturization. In most cases, first-line treatment includes medium- to high-potency topical corticosteroids to control inflammation, accompanied by emollients to reduce transepidermal water loss and support skin healing [1,4]. Antihistamines may be added for symptomatic relief of pruritus [1], while topical calcineurin inhibitors serve as steroid-sparing agents, especially in sensitive areas or for long-term use [4,9]. In severe, extensive, or refractory cases, particularly when systemic involvement is suspected, as in DRESS or HES, systemic corticosteroids or immunosuppressive agents may be required [6,8,9]. Treatment should be tailored to the patient’s clinical presentation, comorbidities, and histopathologic findings to ensure resolution and prevent recurrence. 

## Conclusions

This case underscores the clinical significance of recognizing SD as a histopathologic pattern rather than a discrete diagnosis. Because spongiotic changes are not disease-specific, accurate interpretation requires close correlation of histologic findings with the clinical context. The clinical and histopathological features of SD overlap significantly with other eosinophilic dermatoses, drug reactions such as DRESS, parasitic infections, and HES. These conditions can present with similar rashes, tissue eosinophilia, and even systemic symptoms, requiring a high index of suspicion and broad diagnostic workup.

The patient’s history of travel to Argentina further complicated the evaluation, as parasitic infections are a well-established cause of chronic eosinophilia with cutaneous manifestations. While corticosteroid therapy provided temporary relief, the absence of a confirmed etiology leaves the patient at risk for recurrence or systemic complications. This case reinforces the importance of recognizing SD as a potential mimicker and the need for a multidisciplinary approach, i.e., engaging dermatology, allergy/immunology, infectious disease, and internal medicine, to guide appropriate testing, establish the underlying cause, and ensure long-term management. Unlike previously published reports that focus primarily on isolated dermatologic presentations, this case emphasizes the added complexity of international travel and systemic eosinophilia, expanding the spectrum of diagnostic considerations and underscoring the global relevance of SD as a histopathologic finding.
